# Hypothesis testing for evaluating a multimodal pattern recognition framework applied to speaker detection

**DOI:** 10.1186/1743-0003-5-11

**Published:** 2008-03-27

**Authors:** Patricia Besson, Murat Kunt

**Affiliations:** 1 Signal Processing Institute (ITS), Ecole Polytechnique Fédérale de Lausanne (EPFL), 1015 Lausanne, Switzerland

## Abstract

**Background:**

Speaker detection is an important component of many human-computer interaction applications, like for example, multimedia indexing, or ambient intelligent systems. This work addresses the problem of detecting the current speaker in audio-visual sequences. The detector performs with few and simple material since a single camera and microphone meets the needs.

**Method:**

A multimodal pattern recognition framework is proposed, with solutions provided for each step of the process, namely, the feature generation and extraction steps, the classification, and the evaluation of the system performance. The decision is based on the estimation of the synchrony between the audio and the video signals. Prior to the classification, an information theoretic framework is applied to extract optimized audio features using video information. The classification step is then defined through a hypothesis testing framework in order to get confidence levels associated to the classifier outputs, allowing thereby an evaluation of the performance of the whole multimodal pattern recognition system.

**Results:**

Through the hypothesis testing approach, the classifier performance can be given as a ratio of detection to false-alarm probabilities. Above all, the hypothesis tests give means for measuring the whole pattern recognition process effciency. In particular, the gain offered by the proposed feature extraction step can be evaluated. As a result, it is shown that introducing such a feature extraction step increases the ability of the classifier to produce good relative instance scores, and therefore, the performance of the pattern recognition process.

**Conclusion:**

The powerful capacities of hypothesis tests as an evaluation tool are exploited to assess the performance of a multimodal pattern recognition process. In particular, the advantage of performing or not a feature extraction step prior to the classification is evaluated. Although the proposed framework is used here for detecting the speaker in audiovisual sequences, it could be applied to any other classification task involving two spatio-temporal co-occurring signals.

## Background

Speaker detection is an important component of many human-computer interaction applications, like for example, multimedia indexing, or ambient intelligent systems (through the use of speech-based user-interfaces). Recent and reliable speech recognition methods rely indeed on both acoustic and visual cues to perform [1]. They require therefore the speaker to be identified and discriminated from other users or background noise. The advantage of these interfaces, and what make them appealing for ambient assisted living systems [2], is that they allow to communicate with users in a natural way. This is of course conditioned to the use of simple material for the system to remain light.

The work presented in this paper addresses the problem of detecting the current speaker among two candidates in an audio-video sequence using simple material, namely, a single camera and microphone. A mono audio signal contains no spatial information about the source location, nor does the video signal alone permits to discriminate between a speaker and a person moving his lips – if chewing a gum for example. Therefore, the detection process has to consider both the audio and video cues as well as their inter-relationship to come up with a decision. In particular, previous works in the domain have shown that the evaluation of the synchrony between the two modalities, interpreted as the degree of mutual information between the signals, allowed to recover the common source of the two signals, that is, the speaker [3,4]. Other works, such as [5] and [6], have pointed out that fusing the information contained in each modality at the feature level can greatly help the classification task: the richer and the more representative the features, the more effcient the classifier. Using an information theoretic framework based on [5] and [6], audio features specific to speech are extracted using the information content of both the audio and video signals as a preliminary step for the classification. This feature extraction step is followed by a classification step, where a label "speaker" or "non-speaker" is assigned to pairs of audio and video features. Whereas we have already described in details the feature extraction step in [7] and [8], the classification step is defined here in a new way and constitutes the core contribution of this work.

As stated previously, the classifier decision should rely on an evaluation of the synchrony between pairs of audio and video features. In [6], the authors formulate the evaluation of such a synchrony as a binary hypothesis test asking about the dependence or independence between the two modalities. Thus, a link can be found with mutual information which is nothing else than a metric evaluating the degree of dependence between two random variables [9]. The classifier in [6] ultimately consists in evaluating the difference of mutual information between the audio signal and video features extracted from two potential regions of the image. The sign of the difference indicates the video speech source. We have taken a similar approach in [8], showing, through comparisons with state-of-the-art results, that such a classifier fed with the previously optimized audio features leads to good results.

In the present work, the classification task is cast in a hypothesis testing framework as well. However, the objective – thus, the novelty – is to define not only a classifier, but the means for evaluating the multimodal classification chain – or pattern recognition process – performance. To this end, the hypothesis tests are defined using the Neyman-Pearson frequentist approach [10] and one test is associated to each potential mouth region. This way, the ability of the classifier to produce good relative instance scores can be measured. Moreover, an evaluation of the whole pattern recognition process, including the feature extraction step, can be introduced. It allows to assess the benefit of optimizing features prior to performing the classification.

As a result, a complete multimodal pattern recognition process is proposed in this work, with solutions given for each step of the process, namely, the feature generation and extraction steps, the classification, and finally, the evaluation of the system performance.

## Extraction of optimized audio features for speaker detection: information theoretic approach

Given different mouth regions extracted from an audio-video sequence and corresponding to different potential speakers, the problem is to assign the current speech audio signal to the mouth region which effectively did produce it. This is therefore a decision, or classification, task.

### Multimodal feature extraction framework

Let the speaker be modelled as a bimodal source *S *emitting jointly an audio and a video signal, *A *and *V*. The source *S *itself is not directly accessible but through these measurements. The classification process has therefore to evaluate whether two audio and video measurements are issued from a common estimated source $\hat{S}$ or not, in order to estimate the class membership of this source. This class membership, modeled by a random variable *C *defined over the set Ω_*C*_, can be either "speaker" or "non-speaker". Obviously, the overall goal of the classification process is to minimize the classification error probability *P*_*E *_= *P *($\hat{C}$ ≠ *C*), where the wrong class is assigned to the audio-visual feature pair. In the present case, a good estimation of the class $\hat{C}$ of the source implies a correct estimation $\hat{S}$ of this source. Thus it implies to minimize the probability *P*_*e *_= *P *($\hat{S}$ ≠ *S*) of committing an error during the estimation. The source estimate is inferred from the audio and video measurements by evaluating their shared quantity of information. However, these measurements are generally corrupted by noise due to independent interfering sources so that the source estimate and thus the classifier performance might be poor.

**Figure 1:**
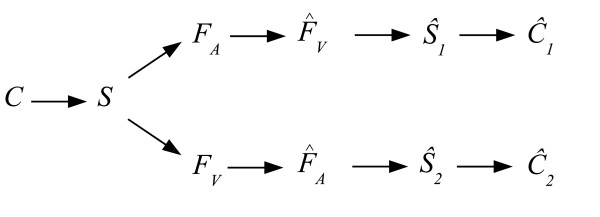
**Classification process**. Graphical representation of the related Markov chains which model the multimodal classification process.

Preliminarily to the classification, a feature extraction step should be performed in order to possibly retrieve the information present in each modality that originates from the common source *S *while discarding the noise coming from the interfering sources. Obviously, this objective can only be reached by considering the two modalities together. Now, given that such features *F*_*A *_and *F*_*V *_(viewed hereafter as random variables defined on sample spaces $\Omega_{F_{A}}$ and $\Omega_{F_{V}}$) can be extracted, the resulting multimodal classification process is described by two first order Markov chains, as shown on Fig. 1[8]. Notice that for the sake of the explanation, the fusion at the decision or classifier level for obtaining a unique estimate $\hat{C}$ of the class is not represented on this graph. *F*_*A *_and *F*_*V *_describe specifically the common source and are then related by their joint probability *p*(*F*_*A*_, *F*_*V*_). Thus, an estimate ${\hat{F}}_{V}$ of *F*_*V*_, respectively, ${\hat{F}}_{A}$ of *F*_*A*_, can be inferred from *F*_*A*_, respectively, *F*_*V *_. This allows to define the transition probabilities for *F*_*A *_→ ${\hat{F}}_{V}$ and *F*_*V *_→ ${\hat{F}}_{A}$ (since *p*(${\hat{F}}_{V}$ |*F*_*A*_) = *p*(${\hat{F}}_{V}$, *F*_*A*_)/*p*(*F*_*A*_), and *p*(${\hat{F}}_{A}$|*F*_*V*_) = *p*(${\hat{F}}_{A}$, *F*_*V*_)/*p*(*F*_*V*_)). Two estimation error probabilities and their associated lower bounds can be defined for these Markov chains, using Fano's inequality and the data processing inequality [5,8]:

(1) $$p_{e_{1}}\geq \frac{H(S)-I(F_{A},{\hat{F}}_{V})-1}{\log\left|\Omega_{S}\right|},$$

(2) $$p_{e_{2}}\geq \frac{H(S)-I(F_{V},{\hat{F}}_{A})-1}{\log\left|\Omega_{S}\right|},$$

where |Ω_*S*_| is the cardinality of *S*, *I *the mutual information, and *H *the entropy. Since the probability densities of ${\hat{F}}_{A}$ and *F*_*A*_, respectively ${\hat{F}}_{V}$ and *F*_*V*_, are both estimated from the same data sequence *A*, respectively *V*, it is possible to introduce the following approximations:

*I*(*F*_*A*_, ${\hat{F}}_{V}$) ≈ *I*(${\hat{F}}_{A}$, *F*_*V*_) ≈ *I*(*F*_*A*_, *F*_*V*_). Moreover, the symmetry property of mutual information allows to define a joint lower bound on the classification error *P*_*e*_:

(3) $$P_{e}=p_{\left\{e_{1},e_{2}\right\}}\geq \frac{H(S)-I(F_{A},F_{V})-1}{\log\left|\Omega_{S}\right|}.$$

To be effcient, the minimization of *P*_*e *_should include the minimization of its associated lower bound. This is done by minimizing the right-hand term of inequality (3), that is, by introducing a constraint on the feature extraction step since it requires to maximize the mutual information between the extracted features *F*_*A *_and *F*_*V *_. In order to both decreases the lower bound on *P*_*e *_and try to get as close as possible to this bound, a mutual information based estimator denoted effciency coeffcient [5,8], is finally defined:

(4) $$e(F_{A},F_{V})=\frac{I(F_{A},F_{V})}{H(F_{A},F_{V})}\in [0,1].$$

Maximizing *e*(*F*_*A*_, *F*_*V*_) still minimizes the lower bound on the error probability defined in Eq. (3) while constraining inter-feature independence. In other words, the extracted features *F*_*A *_and *F*_*V *_will tend to capture specifically the information related to the common origin of *A *and *V*, discarding the unrelated interference information. The interested reader is referred to [8] for more details.

Applying this framework to extract features, we expect to minimize the probability of estimation error. However, to minimize the probability *P*_*E *_of classification error, the last step leading from $\hat{S}$ to $\hat{C}$ must be considered as well. This part deals with the definition of a suitable classifier and will be discussed later on.

### Signal representation

Before applying the optimization framework previously described to the problem at hand, both audio and video signals have to be represented in a suitable way. Notice that the representation chosen here does not need to be the most optimal since an automatic feature optimization step follows.

Physiological evidence points out the motion in the mouth region as a visual clue for speech. It is estimated using the Horn and Schunck gradient-based optical flow [11]. This method leads to a pixel-based representation of the motion and can then capture the complex motions of non-rigid structures like the mouth. To cope with the curse of dimensionality, one-dimensional (1D) video features are preferred. The latter consist finally in the magnitude of the optical flow estimated over T frames in the mouth regions (rectangular regions of size *N *× *M *pixels, including the lips and the chin), signed as the vertical velocity component. The mouth regions are roughly extracted using the face detector depicted in [12]. The set of {*f*_*v*, *n*_}_*n *= 1, ... *N *× *M *× (*T*-1) _observations of the video feature forms the sample of the 1D random variable *F*_*V *_.

Mel-frequency cepstrum coeffcients (MFCCs), widely used in the speech processing community, have been chosen for the audio representation. They describe the salient aspects of the speech signal, while being robust to variations in speaker or acquisition conditions [13]. The mel-cepstrum is downsampled to the video feature rate, so that we finally use a set of *T *- 1 vectors ${\vec{C}}_{t}$, each containing *P *MFCCs:

{*C*_*t*_(*i*)}_*i *= 1,...,*P *_with *t *= 1, ..., *T *- 1 (the first coeffcient has been discarded as it pertains to the energy).

### Audio feature optimization

The information theoretic feature extraction previously discussed is now used to extract audio features that compactly describe the information common with the video features. For that purpose, the 1D audio features *f*_*a*,*t*_($\vec{\alpha }$), associated to the random variable *F*_*A *_are built as the linear combination of the *P *MFCCs:

(5) $$\begin{array}{cc} f_{a,t}(\vec{\alpha })=\sum_{i=1}^{P}\vec{\alpha }(i)\cdot C_{t}(i) & \forall t=1,...,T-1. \end{array}$$

Thus, the set of (*T *- 1) *P*-dimensional observations is reduced to (*T *- 1) 1D values *f*_*a*,*t*_($\vec{\alpha }$). The optimal vector $\vec{\alpha }$ could be obtained straightaway by minimizing the effciency coeffcient given by Eq. (4). However, a more specific and constraining criterion is introduced here. This criterion consists in the squared difference between the effciency coeffcient computed in two mouth regions (referred to as *M*_1 _and *M*_2_). This way, the discrepancy between the marginal densities of the video features in each region are taken into account. Moreover, only one optimization is performed for two mouths resulting in a single set of optimized audio features. It implies however that the potential number of speakers is limited to two in the test audio-video sequences. If $F_{V_{1}}$ and $F_{V_{2}}$ denote the random variables associated to regions *M*_1 _and *M*_2 _respectively, then the optimization problem becomes:

(6) $${\vec{\alpha }}_{opt}=\arg\underset{\vec{\alpha }}{\max}\{{\left[e(F_{V_{1}},F_{A}(\vec{\alpha }))-e(F_{V_{2}},F_{A}(\vec{\alpha }))\right]}^{2}\}.$$

The probability density functions required in the estimation of the mutual information are estimated in a non-parametric way using Parzen windowing. A global optimization method such as an Evolutionnary Algorithm can finally be used to find the optimal set of weights $\vec{\alpha }$[8].

## Hypothesis testing as a classifier and an evaluation tool

The previous section has shown how features specific to the classification problem at hand can be extracted through a multimodal information theoretic framework. The application of this framework results in decreasing the estimation error probability. But the question of minimizing the probability *P*_*E *_of committing an error on the whole classification process still remains. It relies on the choice of a classifier able to classify the extracted features as correctly as possible.

### Hypothesis testing for classification

Hypothesis tests are used in detection problems in order to take the most appropriate decision given an observation x of a random variable *X*. In the problem at hand, the decision function has to decide whether two measurements *A *and *V *(or their corresponding extracted features *F*_*A *_and *F*_*V*_) originate from a common bimodal source *S *– the speaker – or from two independent sources – speech and video noise. As previously stated, the problem of deciding between two mouth regions which one is responsible for the simultaneously recorded speech audio signal can be solved by evaluating the synchrony, or dependence relationship, that exists between this audio signal and each of the two video signals.

From a statistical point of view, the dependence between the audio and the video features corresponding to a given mouth region can be expressed through a hypothesis framework, as follows:

*H*_0 _: *f*_*a*_, *f*_*v *_~ *P*_0 _= *P *(*f*_*a*_) · *P *(*f*_*v*_),

*H*_1 _: *f*_*a*_, *f*_*v *_~ *P*_1 _= *P *(*f*_*a*_, *f*_*v*_).

*H*_0 _postulates the data *f*_*a *_and *f*_*v *_to be governed by a probability density function stating the independence of the video and audio sources. The mouth region should therefore be labeled as "non-speaker". Hypothesis *H*_1 _states the dependence between the two modalities: the mouth region is then associated to the measured speech signal and classified as "speaker". The two hypothesis are obviously mutually exclusive. In the Neyman-Pearson approach [10] certain probabilities associated with the hypothesis test are formulated. The false-alarm probability *P*_*FA*_, or size *α *of the test, is defined as:

(7) $$\alpha =P(\hat{H}=H_{0}|H=H_{1}),$$

while the detection probability *P*_*D*_, or power *β *of the test, is given by:

(8) $$\beta =P(\hat{H}=H_{1}|H=H_{1}).$$

The Neyman-Pearson criterion selects the most powerful test of size *α*: the decision rule should be constructed so that the probability of detection is maximal while the probability of false-alarm do not exceed a given value *α*. Using the log-likelihood ratio, the Neyman-Pearson test can be expressed as follows:

(9) $$\Lambda (f_{a},f_{v})=\log\left[\frac{p(f_{a},f_{v})}{p(f_{a})\cdot p(f_{v})}\right]⋛\eta ,$$

The test function must then decide which of the hypothesis is the most likely to describe the probability density functions of the observations *f*_*a *_and *f*_*v*_, by finding the threshold *η *that will give the best test of size *α*.

The mutual information is a metric evaluating the distance between a joint distribution stating the dependence of the variables and a joint distribution stating the independence between those same variables:

(10) $$I(F_{A},F_{V})=\sum_{f_{a}\in \Omega_{F_{A}}}\sum_{f_{v}\in \Omega_{F_{V}}}\left[p(f_{a},f_{v})\log\left(\frac{p(f_{a},f_{v})}{p(f_{a})\cdot p(f_{v})}\right)\right].$$

The link with the hypothesis test of Eq. (7) seems straightforward. Indeed, as the number of observations *f*_*a *_and *f*_*v *_grows large, the normalized log-likelihood ratio approaches its expected value and becomes equal to the mutual information between the random variables *F*_*A *_and *F*_*V *_[9]. The test function can then be defined as a simple evaluation of the mutual information between audio and video random variables, with respect to a threshold *η*. This result differs from the approach of Fisher *et al*. in [6], where the mouth region which exhibits the largest mutual information value is assumed to have produced the speech audio signal. The formulation of the hypothesis test with a Neyman-Pearson approach allows to define a measure of confidence on the decision taken by the classifier, in the sense that the *α*-*β *trade-off is known. Considering that two mouth regions could potentially be associated to the current audio signal and defining one hypothesis test (with associated thresholds *η*_1 _and *η*_2_) for each of these regions, four different cases can occur:

1. *I*_1_(*F*_*A*_, $F_{V_{1}}$) > *η*_1 _and *I*_1_(*F*_*A*_, $F_{V_{2}}$) <*η*_2_: speaker 1 is speaking and speaker 2 is not;

2. *I*_1_(*F*_*A*_, $F_{V_{1}}$) <*η*_1 _and *I*_1_(*F*_*A*_, $F_{V_{2}}$) > *η*_2_: speaker 2 is speaking and speaker 1 is not;

3. *I*_1_(*F*_*A*_, $F_{V_{1}}$) <*η*_1 _and *I*_1_(*F*_*A*_, $F_{V_{2}}$) <*η*_2_: none of the speaker is speaking;

4. *I*_1_(*F*_*A*_, $F_{V_{1}}$) > *η*_1 _and *I*_1_(*F*_*A*_, $F_{V_{2}}$) > *η*_2_: both speakers are speaking.

The experimental conditions are defined so as to eliminate the possibilities 3 and 4: the test set is composed of sequences where speakers 1 and 2 are speaking each in turn, without silent states. This allows, in the context of this preliminary work, to define the simpler following cases: if a speaker is silent, it implies that the other one is actually speaking. Notice also that a possible equality with the threshold is solved by attributing randomly a class to the random variable pair.

### Hypothesis testing for performance evaluation

The formulation of the previous hypothesis test gives means for evaluating the whole classification chain performance. Receiver Operating Characteristic (ROC) graphs allow to visualize and select classifiers based on their performance [14]. They permit to crossplot the size and power of a Neyman-Pearson test, thus to evaluate the ability of a classifier to produce good relative instance scores. Our purpose here is not to focus only on the evaluation on the classifier itself but on the possible gain offered by the introduction of the feature optimization step in the complete pattern recognition process. To this end, two kinds of audio features are used in turn to estimate the mutual information in each mouth region: the first ones are the linear combination of the MFCCs resulting from the optimization described previously; the second ones consist simply in the mean value of these MFCCs. The results about this comparison are presented in the next section.

## Results

Firstly, the ability of hypothesis testing to act as a classifier is discussed. The evaluation of the possible gain offered by using optimized audio features with respect to simpler ones is addressed next.

### Experimental protocol

The sequence test set is composed of the eleven two-speaker sequences *g11 *to *g22 *taken from the CUAVE database [15], where each speaker utters in turn two digit series (notice that *g18 *has been discarded as it exhibits strong noise due to the compression). These sequences are shot in the NTSC standard (29.97 fps, 44.1 kHz stereo sound). For the purpose of the experiments, the problem has been restricted to the case where one of the speaker and only one of them is speaking in any case. Therefore, the last seconds of the video clips where the two speakers are speaking all together, as well as the silent frames – labelled as in [16] – have been discarded.

**Figure 2:**
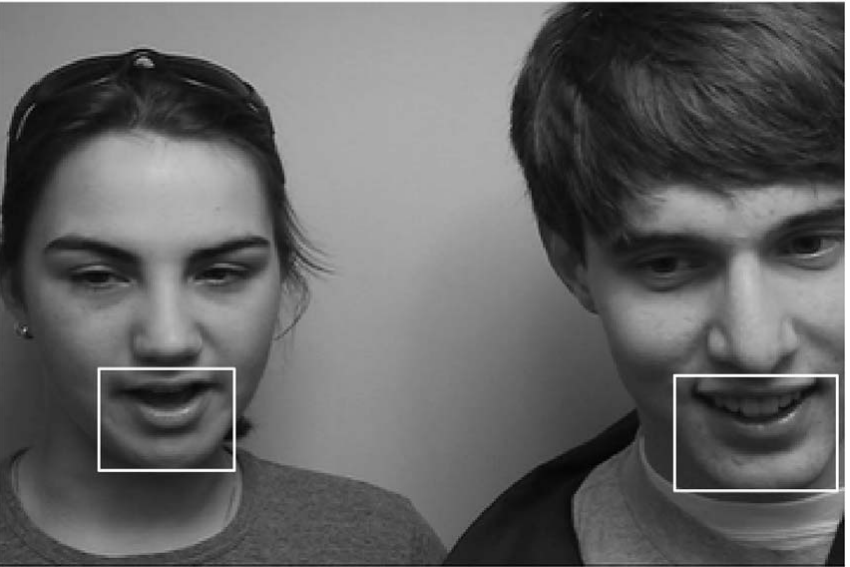
**Frame example from the CUAVE database**. Frame example taken from the sequence g13 of the CUAVE database [15]. The white boxes delimited the extracted mouth regions.

For all the sequences, the *N *× *M *mouth regions are extracted, using the face detector given in [12] (*N *and *M *varying between 30 and 60 pixels, depending on speakers' characteristics and acquisition conditions). A frame example taken from the CUAVE database is shown in Fig. 2, together with the corresponding extracted mouth regions (white boxes).

The video feature set is composed of the *N *× *M *× (*T *- 1) values of the optical flow norm at each pixel location (T being the number of video frames within the analyzing window, *i.e*. *T *= 60 frames). From the audio signal, 12 mel-cepstrum coeffcients are computed using 30 ms Hamming windows.

The optimization is done over a 2 second temporal window, shifted by one second steps over the whole sequence to take decisions every seconds. The output of the classifier for each window is compared to the corresponding ground truth label, defined as in [16]. The test set is eventually composed of 188 test points (windows), with one audio and one video instances for each window. The two classes, "speaker1" (speaker on the left of the image) and "speaker2" (speaker on the right) are well balanced since theirs set sizes are 95 and 93 respectively.

### Performance of hypothesis testing as a classifier

The classifier is defined as the test function giving the best test of size *α *and receives the optimized audio features at input.

For binary tests, a positive and a negative class have to be defined. We assume the positive class to be the class "speaker" for each test. More precisely, since the experimental conditions implies that there is always one speaker speaking, the positive class is the label of the mouth region where the test is performed: *i.e*, "speaker1" for test1 (defined between the random variables *F*_*A *_and *F*_*V*1_), and "speaker2" for test2. Table 1 compares the power of the tests for given sizes *α*.

**Table 1:** Power of the tests for given sizes. Power *β *of the tests for different sizes *α*. The thresholds *η *defining the corresponding decision functions are also indicated.

	Test1			Test2		
		
*α*	5%	10%	20%	5%	10%	20%
*β*	37.9%	81.1%	90.5%	4.3%	24.7%	89.26%
*η*	0.41	0.25	0.16	0.55	0.45	0.25

Let us introduce now the accuracy of a test as the sum of the true positive and true negative rates divided by the total number of positive and negative instances [14]. Table 2 gives the classifier scores for the threshold corresponding to each test best accuracy: 86.7% and 85.11% for test1 and test2 respectively, obtained for thresholds *η*_1 _= 0.18 and *η*_2 _= 0.19.

**Table 2:** *β *and *α *for best accuracy values. Power *β *and size *α *for each class of each test at its best accuracy value.

	Test1		Test2	
		
	Positive class	Negative class	Positive class	Negative class
		
*β*	87.4%	86.0%	91.4%	79.0%
*α*	14.0%	12.6%	21.0%	8.6%

These results indicate hypothesis test as a good method for assigning a speaker class to mouth regions, with a given *α*-*β *trade-off (thus greater adaptability to changes of the target condition or the classification requirement). The classifier produces better relative instance scores for test1. However, the thresholds giving the best accuracy values are about the same for the two tests. This tends to indicate that this threshold is not speaker dependent. Further tests on larger test sets would be necessary however for a more precise analysis of the classifier capacity.

### Evaluation of the pattern recognition process performance

**Figure 3:**
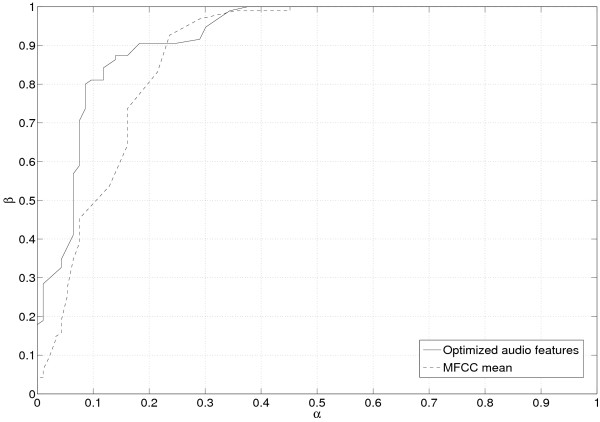
**ROC graph for test1**. ROC graph for test 1. The detection probability for the positive class is plotted versus the false-alarm rate.

**Figure 4:**
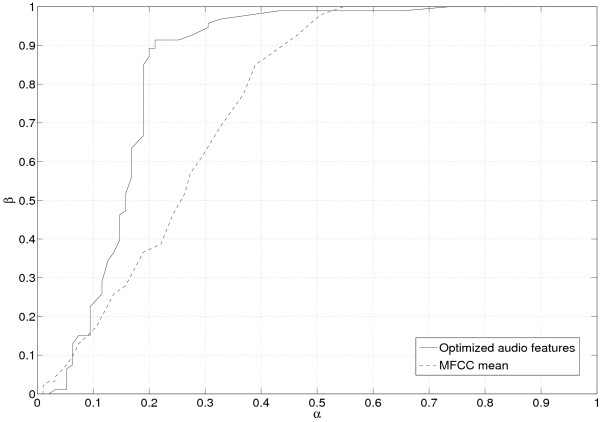
**ROC graph for test2**. ROC graph for test 2. The detection probability for the positive class is plotted versus the false-alarm rate.

The advantage of using optimized audio features against simple ones at the input of the classifier is now discussed. As in the previous paragraph, two tests are considered, with the positive classes being respectively the "speaker 1" and the "speaker 2". The ROC graphs corresponding to each test are plotted on Figs. 3 and 4. An analysis of these curves shows that the classifier fed in with the optimized audio features performs better in the conservative region of the graph (northwest region).

Table 3 sums up some interesting values attached to the ROC curve such as the area under the curve (AUC), or the accuracy with corresponding thresholds. Whatever the way of considering the problem, the use of the optimized audio features improved the classifier average performance, as stated by the theory.

**Table 3:** Area under the curves. Area under the curve and accuracy with the corresponding threshold *η *for each test.

	Test 1		Test 2	
		
Input features	MFCCs mean	Optimized audio features	MFCCs mean	Optimized audio features
		
AUC	0.88	0.92	0.75	0.84
Accuracy	84, 6%	86, 7%	73, 4%	85, 1%
*η*	0.14	0.18	0.10	0.19

## Conclusion

This work addresses the problem of labeling mouth regions extracted from audio-visual sequences with a given speaker class label. The system uses a simple material, namely a single microphone and camera. The detector must then analyze jointly the audio and video information to come to a decision. The problem is cast in a hypothesis testing framework, linked to information theory. The resulting classifier is based on the evaluation of the mutual information between the audio signal and the mouths' video features with respect to a threshold, issued from the Neyman-Pearson lemma. A confidence level can then be assigned to the classifier outputs. This allows firstly to adapt the classifier to changes of the target condition or of the classification requirement. Secondly, this approach results in the definition of an evaluation framework. The latter is not only used to determine the performance of the classifier itself, but considers rather rating the whole pattern recognition process effciency.

In particular, it is used to check whether a feature extraction step performed prior to the classification can increase the accuracy of the detection process. Optimized audio features obtained through an information theoretic feature extraction framework feed the classifier, in turn with non-optimized audio features. Analysis tools derived from hypothesis testing, such as ROC graphs, establish eventually the performance gain offered by introducing the feature extraction step in the process.

As far as the classifier itself is concerned, more intensive tests should be performed in order to draw robust conclusions. However, preliminary remarks tend to indicate that a hypothesis-based model can be used with advantage for multimodal speaker detection. It would also be interesting to consider in future works the cases of simultaneous silent or speaking states (cases 3 and 4 defined previously).

As a final remark, let us stress that the multimodal pattern recognition framework we propose does not apply exclusively to speaker detection. It can be used with advantage for other applications, provided bimodal signals co-occurring in space and time are involved. One might think for example to medical applications where several synchronized biological signals exist and are to be processed to come to a diagnostic.

## Competing interests

The author(s) declare that they have no competing interests.

## Authors' contributions

A complete multimodal pattern recognition approach has been proposed. It is applied here for detecting the speaker in audio-video sequences but could be applied to other pattern recognition tasks involving bimodal signals co-occurring in space and time. An information theoretic feature extraction is performed prior to the classification. The definition of the classification step through a hypothesis testing framework is the main contribution of this work. It completes the pattern recognition process as it gives means for evaluating the performance of the classifier as well as of the whole pattern recognition process.
